# Multiple Sclerosis and HERV-W/MSRV: A Multicentric Study

**Published:** 2007-12

**Authors:** Giannina Arru, Giuseppe Mameli, Vito Astone, Caterina Serra, Yu-Min Huang, Hans Link, Enrico Fainardi, Massimiliano Castellazzi, Enrico Granieri, Miriam Fernandez, Pablo Villoslada, Maria Laura Fois, Alessandra Sanna, Giulio Rosati, Antonina Dolei, Stefano Sotgiu

**Affiliations:** 1 *Institute of Clinical Neurology, University of Sassari, Viale San Pietro 10, 07100, Sassari, Italy;*; 2 *Department of Biomedical Sciences, University of Sassari, Viale San Pietro 43/B, 07100 Sassari, Italy;*; 3 *Neurotec Department, Karolinska Institute,Alfred Nobels Allé 10, 141 83 Stockholm, Sweden;*; 4 *Department of Neurology, University of Ferrara, Corso della Giovecca 203, Ferrara I-44100, Italy;*; 5 *Department of Neurology, Clinica Universitaria de Navarra, Pio XII 36, 31008, Pamplona, NA, Spain*

**Keywords:** human endogenous retrovirus, HERV-W, MSRV, multiple sclerosis

## Abstract

We designed a large multicentric study to analyse the presence of MSRV particles in blood and CSF of a large cohort of patients and controls from different European areas. 149 MS patients and 153 neurological and healthy controls were selected from Sardinia, Spain, Northern-Italy and Sweden. To avoid biological and inter-assay variability MSRV was detected within a single laboratory through nested and real-time PCR assays specific for *pol* and *env* genes. MSRV detection in blood and CSF of MS patients and controls in populations of different ethnicity gave significant differences (*p*<0.05 compared to neurological controls and <0.001 compared to healthy controls). The presence and viral load of MSRV are significantly associated with MS as compared to neurological and healthy controls in all ethnic groups.

## INTRODUCTION

Human endogenous retrovirus (HERV) family accounts for at least 8% of the whole human genome. In general, most of them are highly defective, but a few cases of complete proviruses have been described (1). The founder member of the human endogenous retroviruses (HERV)-W family, originally discovered in multiple sclerosis (MS) patients (2, 3), has been designated as MS-associated retrovirus (MSRV) (4, 5). RNA pol-sequences of MSRV have been confirmed in MS by subsequent independent studies (6-9), related to MS severity (10-12), and brain pathogenicity (13). HERV-W *gag* and *env* protein have been shown to be increased in MS brains (14) and capable of activating Th1-like and innate immunity (15) and CNS demyelination (16). However, other studies suggest HERV-W/MSRV is only casually associated with MS (17, 18). This incongruence may relate to variability in patients’ condition and population genetics, as well as intrinsic methodological differences (7, 11).

With the aim of providing additional evidence ragarding whether HERV-W/MSRV is associated with MS, we assayed for the presence of HERV-W/MSRV particles in blood and cerebrospinal fluid (CSF) of MS patients and controls in a very large cohort of patients and controls from different European areas. Uniform patient selection and polarisation of the HERV-W/MSRV detection were adopted to limit biologic and methodological variables.

## MATERIAL AND METHODS

**Figure 1:**
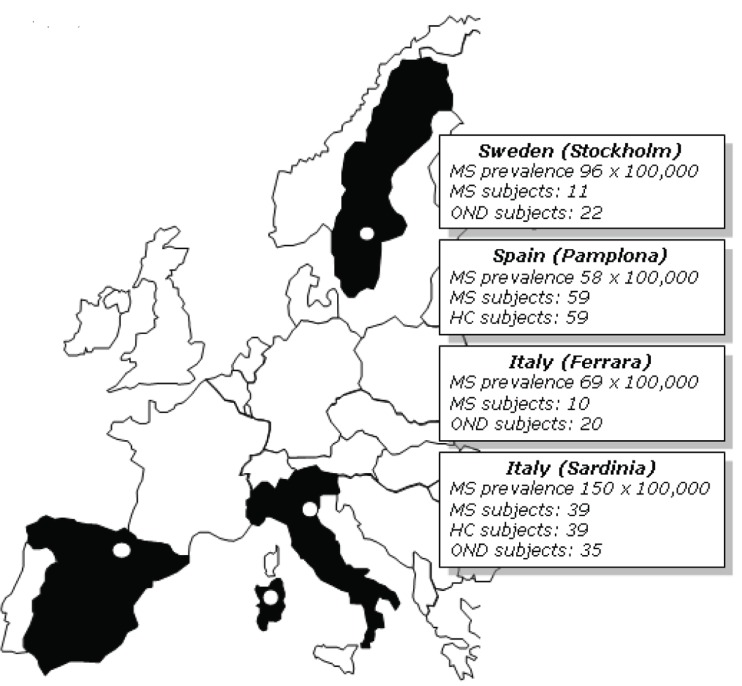
Study composition and mean MS prevalence rates (× 100,000 population^17^) in the four European areas under the present investigation.

One-hundred-forty-nine (149) MS patients and 153 controls were selected from four distinct European areas: Sardinia (insular Italy), Ferrara (Northern Italy), Pamplona (Spain) and Stockholm (Sweden), each with its own peculiar mean MS prevalence rate (19, Figure 1). All donors gave informed consent to the study.

### Sardinians

Thirty-nine active untreated MS patients diagnosed according to the Poser criteria (20) (mean age 36.7 years; woman/man ratio 1.9) were selected; 39 age- and sex-matched healthy subjects and 35 patients with other neurological diseases (OND) were also selected. OND controls included inflammatory post-infectious encephalomyelitis (2 patients), CNS vasculitis (3), and non inflammatory neurological diseases including migraine, compressive radiculo-myelopathy, amyotrophic lateral sclerosis, heredo-degenerative and toxic peripheral neuropathy.

### Northern Italians (Ferrara)

CSF samples from 10 untreated MS patients (20) (4 women, mean age 39.4, range 27-67) were collected at the Institute of Neurology, University of Ferrara. CSF samples from 20 OND patients (8 women, mean age 51.2 years) served as control including migraine, peripheral neuropathy, amyotrophic lateralsclerosis, heredo-degenerative ataxia and 5 patients with CNS vasculitis.

### Spanish (Pamplona, Navarra)

Fifty-nine blood samples from untreated relapsing and secondary progressive MS patients (20) (42 women, mean age 31.8) and 59 age-and sex-matched Spanish HC were collected at the Department of Neurology, University of Navarra.

### Swedish (Stockholm)

Forty-nine patients (35 women, mean age 34 years) with acute monosymptomatic optic neuritis (ON) were consecutively enrolled at the Department of Ophthalmology, Huddinge University Hospital. Every six months, all patients were subjected to detailed history and neuro-ophthalmologic examination (11). Eleven such patients developed CDMS (20). HERV-W/MSRV from blood and CSF samples of this ON sub-cohort were analysed. Twenty-two non inflammatory OND individuals (mean age 49.5 years) were examined as control including headache, Bell’s palsy, vertigo, hydrocephalus, polyneuropathy and sensory disturbances of unknown origin.

### Detection of extracellular MSRV

To avoid potentially biasing methodological differences between laboratories, all of the molecular assays were performed at the Department of Biomedical Sciences, University of Sassari. All samples coming from Stockholm, Pamplona, Ferrara and Neurology Clinic of Sassari were transported frozen, handled identically after freezing and tested concurrently under strict blind code.

**Nested RT-PCR of *pol* product.** This method has been extensively reported in other publications from our group (7).

**Real-time RT-PCR:** RNA extraction of 500 ml plasma/CSF samples and reverse transcription by using oligo-dT as primer PCR were carried out in a 25 ml volume containing 1X PCR buffer (Bio-Rad Hercules, CA), 1.5 mmol/L MgCl_2_, 200 nmol/L of each primer, 200 mmol/L of dNTPs, and 100 nmol/L of TaqMan probes, 1.25 units of iTAQ DNA polymerase (Bio-Rad Hercules, CA). The TaqMan assay amplification reactions were initially heated at 95°C for 3’ and then subjected to 50 cycles of 94°C for 15 seconds, 53°C for 30”, and 60°C for 30” with iCycler iQ PCR Detection System (Bio-Rad, Hercules, CA). Primers pair ENVF (5’-GCCCCATCGTATAGGAGTCTTTC-3’ bases 1188-1210) and ENVR (5’-AGTGGCAGAGTGATAGCAGTTG-3’ bases 1253-1274) and TaqMan probes ENVP FAM-5’-CCCACCTTCACTGCCCACACCCAT-3’-TAMRA bases 1221-1244 (SIGMA Genosys Ltd UK). They were derived from the *env* gene for HERV-W/MSRV encoded RNA (GenBank accession number GI48949850). The RT-PCR products, (an 87-bp fragment) were cloned in Pcr 2.1 (Invitrogen Carlsbad, CA). Absolute copy number of plasmids were estimated according to the formula: n. molecules/μL= {plasmids concentration (g/l)/ [(fragment size “87 bp” + plasmid size “3931 bp”) × 660]} × 6,022 × 10^23^.

A standard graph of the cycle threshold (*C_T_*) was obtained from serial plasmid dilutions (10 to 10^5^ copies/well). The *C_T_* values from unknown samples were plotted on the respective standard curves, and the number of RNA env gene copies calculated. Each RNA extract and plasmid serial dilutions were analyzed in duplicate. To ensure that the PCR sensitivity did not differ between assays or change over time, and to evaluate the correct amplification, three HERV-W/MSRV positive samples were also tested during each amplification, starting from the same *C_T_*. To control for correct amplification and retrotranscription, a HERV-W/MSRV positive sample was also submitted with each RNA extraction procedure, and the resulting extract amplified in duplicate. To confirm sample RNAs, we performed PCR amplification without RT reaction with pure mRNA samples, indicating that the prepared mRNA samples did not contain genomic DNA. Samples were considered negative if the *C_T_* values exceeded 40 cycles.

### Statistical analysis

The distribution of the nested RT-PCR was analyzed by using the χ^2^. Real-time data were managed by using mean and standard deviation. The unpaired T-test and Fisher’s exact test were used to compare different sample categories. Statistical significance was established for *p* value <0.05. A correlation study was performed to compare the viral load and the rate of HERV-W/MSRV^+^ of MS groups from different countries with the country-specific mean prevalence rate of MS. For this, the Pearson’s correlation coefficient (r) was calculated.

## RESULTS

### Detection of HERV-W/MSRV viraemia in MS and controls by nested PCR

In the whole cohort, HERV-W/MSRV detection through nested RT-PCR (i.e. proportion of HERV-W/MSRV-positive cases) in blood and CSF of MS patients and controls gave significant differences. In particular, the percentage of HERV-W/MSRV^+^ MS patients was 71.4% in blood compared to 17.3% in HC (*p*<0.001) and 40.3% in OND controls (*p*<0.001). In the CSF, 74.5% MS and 45.6% OND had HERV-W/MSRV RNA (*p*<0.001; Table 1).

**Table 1:** Summary of MSRV detection through RT-PCR (A. proportion of MSRV-positive cases) and real-time RT-PCR (B. number of MSRV copies/ml) in blood and CSF of MS patients and controls in the European populations

A. Nested PCR *(pol)*	MS		OND		HC
	Plasma	CFS	Plasma	CSF	Plasma

**ALL**	105/147	73/98	23/57	37/81	17/98
	(71.4%)	(74.5%)	(40.3%*)	(45.6%*)	(17.3%**)

**B. Real-time PCR (*env*)**	**MS**		**OND**		**HC**
	**Plasma**	**CFS**	**Plasma**	**CSF**	**Plasma**

**ALL**	2527	3190	256**	293**	327**

*p* values for plasma and for CSF (MSRV viraemia in active MS vs. OND and HC)=*<0.05; **<0.001.

* <0.05;

** <0.001.

European populations were stratified according to their provenance. Among Sardinians the percentage of HERV-W/MSRV^+^ MS patients was 100% in blood compared to 12.8% in HC (*p*<0.0001) and 42.8% in OND controls (*p*<0.0001). In Sardinian CSF, 82% MS and 57% OND had HERV-W/MSRV RNA (*p*<0.001). Among Northern-Italians, 80% MS patients were MSRV^+^ in CSF compared to 40% OND (*p*=0.02). Among Swedish, the HERV-W/MSRV^+^ rate was 73.4% in blood and 67.3 in CSF of MS compared to 36.4% and 31.8%, respectively, of OND (*p*=0.0005). Among Spanish the rate of HERV-W/MSRV^+^ in blood was 59.3% in MS and 20.3% in HC (*p*=0.01, Table 2).

**Table 2:** Details of the MSRV detection through nested RT-PCR (A. proportion of MSRV-positive cases) and real-time RT-PCR (B. number of MSRV copies/ml) in blood and CSF of MS patients and controls in the European populations

A. Nested PCR*(pol)*	MS		OND		HC
	Plasma	CFS	Plasma	CSF	Plasma

**Sardinia**	39/39 (100%)***	32/39 (82%)***	15/35 (42.8%)	22/39 (57%)	5/39 (12.8%)
**Northern Italy**	-	8/10 (80%)*	-	8/20 (40%)	-
**Spain**	30/59 (59.3%)*	-	-	-	12/59(20.3%)
**Sweden**	36/49 (73.4%)**	33/49 (67.3%)**	8/22 (36.4%)	7/22 (31.8%)	-

**B. Real-time PCR *(env)***	**MS**		**OND**		**HC**
	**Plasma**	**CFS**	**Plasma**	**CSF**	**Plasma**

**Sardinia**	2036***	2650***	180	162	312
**Northern Italy**	-	144*	-	55	
**Spain**	72**	-	-	-	15
**Sweden**	419**	396*	76	76	-

*p* values for plasma and for CSF (MSRV viraemia in active MS vs. OND and HC) are indicated in asterisks: *<0.05; **<0.001; ***<0.0001.

* <0.05;

** <0.001;

*** <0.0001.

### Evaluation of HERV-W/MSRV load in MS and controls by real-time RT-PCR

In summary, HERV-W/MSRV detection through real-time RT-PCR (i.e. number of HERV-W/MSRV copies/ml) in blood and CSF of MS patients and controls in the whole multicentric European series gave significant differences. In blood, MS patients produced an average 2527 ± 2376 SD HERV-W/MSRV copies/ml in plasma compared to 256 ± 152 of OND controls (*p*<0.0001) and 327 ± 173 in HC. As for the CSF, MS patients produced an average 3190 ± 1920 HERV-W/MSRV copies compared to OND (293 ± 221; *p*<0.0001; Table 1).

European populations were therefore stratified according to their provenance. Sardinian MS patients produced an average 2159 ± 2176 SD HERV-W/MSRV copies/ml in plasma relative to 180 ± 110 of OND controls (*p*<0.0001). Spanish MS patients produced 72.3 ± 44.4 HERV-W/MSRV copies/ml in plasma compared to 15 ± 2 of the HC (*p*=0.002). Swedish MS patients produced mean 419.4 ± 109.8 copies/ml in blood compared to 75.9 ± 18.9 of OND (*p*=0.0005). As for the CSF, Sardinian MS patients produced an average 2650 ± 1830 HERV-W/MSRV copies compared to OND (162 ± 112; *p*<0.0001). In Northern-Italians an average 144.2 ± 39 CSF HERV-W/MSRV copies were found in MS patients and 55.3 ± 16.6 in OND controls (*p*=0.02). Among Swedish, 396.3 ± 28.4 is the mean value in MS compared to 76.1 ± 16.9 in OND (*p*=0.04; Table 2).

## DISCUSSION

Similar to other viruses, HERV-W/MSRV has been linked to MS by many groups, but only casually associated by others. Many biological and methodological biases might account for such variability (11). Therefore we designed a multicentric-polarised study to report on the actual association between HERV-W/MSRV and MS.

In all samples that tested positive, we found the presence of both HERV-W/MSRV-*env* and -*pol* RNAs, regardless of the health/disease status of the individual, thus suggesting a co-ordinate accumulation of the two transcripts, as it occurs for closely located genes. As observed in our previous work on the accumulation of MSRV/HERV-W gene transcripts in MS brains (21) the quantitative real-time and the semi-quantitative RT-PCR of *env* and *pol* genes gave comparable results. A statistically significant increase was observed in MS patients compared to controls, with respect to both the percentage of HERV-W/MSRV positivity (nested RT-PCR) and the viral load (quantitative real time RT-PCR) in blood and, when available, in CSF. However, no intra-individual correlations between blood and CSF viral loads were found. We have previously seen by nested-RT-PCR that blood-MSRV^+^ patients may be CSF- and vice versa (7, 10). In the present study, the quantitative analysis (real-time) indicates that the viral load of MSRV is also different between distinct compartments in the same individual.

Overall, our data indicate that in distinct ethnic groups HERV-W/MSRV release is significantly elevated in MS as compared to other controls. In the recent past (7) we found that MSRV positivity in OND was significantly higher (63.6% in blood) in patients with inflammatory OND as compared to patients with non–inflammatory OND (33%, *p*<0.001 in blood). This suggests that MSRV is not specific for MS, despite its acronym. Therefore, HERV-W/MSRV appears to be more suggestive of inflammation rather than merely MS. At the etiopathogenic level, the higher release of the virus in MS patients does not provide definitive evidence that HERV-W/MSRV release is a causal event, nor even a secondary pathogenic co-factor; on the contrary, it may reflect a generic predisposition to it: in fact, HERV-W/MSRV *pol* gene expression is found in 69% sera from South-African MS patients, absent in serum of 39 unrelated HC (*p*<0.001) but present in 70% of the unaffected close relatives of the MS patients (6). Alternatively, HERV-W/MSRV production might reflect an indirect, coincidental effect of the dysfunctional inflammatory-degenerative cascade occurring in MS (22). Th1 detrimental cytokines exert a HERV-W/MSRV-releasing effect on HERV-W/MSRV^+^ monocytes *in vitro*, whilst IFN-β actively down-modulates its expression (23). Perhaps due to this modulation, the pro-inflammatory cytokine pattern which develops in MS may up-modulate HERV-W/MSRV expression, thus allowing speculation on an immune-mediated HERV-W/MSRV release rather than a primary causative effect of HERV-W/MSRV in MS.

Some other points also deserve the same interpretative caution. One is the question of what factors might explain the geographical-ethnic differences in the prevalence of MSRV in MS populations versus control groups. It is known that genetic factors play a pivotal role in MS. One could argue that, as Sardinia is a small geographically isolated island with a very genetically isolated population, some unknown genetic selective pressures could have forced the Sardinian genome to contain thousands copies of MSRV. However, preferential circulation of MSRV in geographically closed population such as Sardinians cannot be excluded.

The clinical groups were dissimilar in terms of sample extension which may rise concerns when making comparisons between countries. However, even though patient selection was not uniform in terms of absolute numbers, the study was set to ensure that all samples were collected and stored at the same time and under similar conditions. Although RNA is prone to degradation, sample transportation has not been likely to have influenced the results since all samples contained 200U of RNAse Inhibitor and were carefully shipped.

In conclusion, the present study confirms HERV-W/MSRV to be an important marker of the ongoing process occurring in MS. With its well known pathogenic potential (1) and its association with a more severe MS course (10-12), HERV-W/MSRV may represent an interesting issue for future patho-physiological and therapeutical studies.
